# The impact of interventions to modify antibiotic use in children with suspected infections in ambulatory healthcare settings in LMICs: a systematic review

**DOI:** 10.1093/jacamr/dlag109

**Published:** 2026-06-13

**Authors:** Jack S Hardman, Gift Chareka, Elizabeth O’Mahony, Admire S Murongazvombo, Christine Chew, Sunanda C Ray, Shunmay Yeung, Felicity C Fitzgerald

**Affiliations:** Infection, Immunity and Inflammation, UCL Great Ormond Street Institute of Child Health, London, UK; Faculty of Medicine and Health Sciences, University of Zimbabwe Clinical Trials Research Centre, Harare, Zimbabwe; Centre for Genomics and Child Health, Blizard Institute, Queen Mary University of London, London, UK; Department of Health, Bindura University of Science Education, Bindura, Zimbabwe; School of Cellular and Molecular Medicine, University of Bristol, Bristol, UK; Department of Medical Education, University of Botswana, Gaborone, Botswana; Clinical Research Department, Faculty of Infectious and Tropical Diseases, London School of Hygiene & Tropical Medicine, London, UK; Department of Paediatric Infectious Diseases, St Mary’s Imperial College London, London, UK; Department of Infectious Disease, School of Medicine, Imperial College London, London, UK; The Health Research Unit Zimbabwe (THRU-Zim), Biomedical Research and Training Institute, Harare, Zimbabwe

## Abstract

**Background and objectives:**

Antimicrobial resistance (AMR) is a growing threat to child health in low- and middle-income countries (LMICs), where inadequate antibiotic access frequently causes morbidity and mortality. We sought to identify effective interventions to optimize antibiotic use in children with suspected infections in LMIC ambulatory healthcare settings.

**Methods:**

MEDLINE, Embase, Cochrane Central Register of Controlled Trials (CENTRAL), Cochrane Infectious Disease Group Specialized Register, Web of Science, PsycInfo, WHO library database (WHOLIS) and regional databases (inception until 16 December 2024) were searched to identify studies of interventions to optimize antibiotic use in children in LMIC peripheral healthcare settings.

**Results:**

Our search identified 7154 articles post de-duplication; 23 studies met the inclusion and Integrated quality Criteria for the Review of Multiple Study designs (ICROMS) assessment criteria. Seventeen were randomized trials, conducted in five LMICs, four upper-middle income countries (UMICs) and two low-income countries (LICs). Single-intervention studies investigated electronic algorithms (*n* = 3) and point-of-care tests (POCTs) (*n* = 4). Electronic algorithms and enhanced POCTs reduced antibiotic prescriptions for children with acute illness at primary care facilities in Tanzania. C-reactive protein POCTs achieved modest reductions in antibiotic prescribing for children in Vietnam and Uganda but not for those with febrile illness in Myanmar and Thailand. A diagnostic tool algorithm showed reduction of antibiotic prescriptions in Ghana and Burkina Faso, but not in Uganda.

**Conclusions:**

Initial evidence suggests that algorithmic decision-support tools combined with point-of-care diagnostics and provider education can reduce antibiotic prescribing in children attending ambulatory healthcare settings in LMICs, with most studies demonstrating no compromise of clinical outcomes. However, few studies assessed prescription appropriateness or microbiological outcomes, limiting conclusions about long-term impact. Future interventions should prioritize context-adaptable strategies that incorporate rapid diagnostics, evaluate clinical and appropriateness outcomes, and consider implementation feasibility across diverse LMIC settings.

## Introduction

Antimicrobial resistance (AMR) poses a critical threat to public health and is associated with an estimated 4.95 million deaths annually.^1^ Low- and middle-income countries (LMICs) are disproportionately affected by AMR, with higher mortality rates from resistant organisms.^1^ Infectious diseases are the major cause of death in children under 5 years of age in LMICs, with inadequate antibiotic access continuing to cause more deaths than AMR.^2^ Although antibiotic use varies significantly between and within LMICs, recent increases in global antibiotic consumption have been driven by rising antibiotic use in LMIC settings.^3,4^

Inpatient antimicrobial stewardship (AMS) interventions have been shown to effectively improve adherence to antibiotic guidelines, reduce duration of antibiotic treatment and reduce the incidence of infections with resistant bacteria, without impacting mortality.^5–7^ AMS interventions have frequently been categorized as enabling (guidelines, education, training), persuasive (audits with feedback to prescribers), restrictive (expert approval required for prescriptions, clinician-issued prescriptions) and structural/decision support (electronic medical record introduction, therapeutics committees).^8,9^

Optimizing antibiotic use is a balance between improving access in cases where they are needed and reducing use where they are not; but the decision on whether they are needed or not is often multifactorial and ambiguous. Antibiotic use in LMICs has increased as healthcare access has widened and drug costs have reduced, reaching two-and-a-half times that of high-income countries.^4^ Despite this, over a million children a year still die of untreated pneumonia and sepsis, demonstrating ongoing access challenges.^2^

Most evidence of interventions to optimize antibiotic use has come from inpatient studies in high-income countries (HICs), where healthcare systems have greater access to adequate funding, diagnostic equipment, microbiology support and infectious disease specialists.^6^ However, the burden of AMR is greatest in LMICs, and most antibiotic consumption occurs in community (or peripheral healthcare) settings, with an estimated 78% of antibiotic use in LMICs described as self-medicated.^1,10,11^ The diversity of healthcare systems and economic, regulatory and cultural differences between LMICs influence where antibiotics are accessed;^12^ for example the use of non-prescribed antibiotics in LMICs is higher in lower socioeconomic settings and most commonly provided by pharmacies, which often have fewer barriers to attending than primary care facilities.^11^ A systematic review on the use of non-prescribed antibiotics found a pooled prevalence of 78% utilization, with pharmacies being the primary source.^11^ Effective interventions to optimize antibiotic use in LMICs must be designed with an understanding of these contextual differences for them to be sustainable and create change.^12^ Interventions must target not only prescription of antibiotics in healthcare settings, but the considerable burden occurring in the community within pharmacies, drug shops and in reused prescriptions.

There have been few systematic reviews of interventions to optimize antibiotic use in children in LMICs. One previous systematic review of hospital and community AMS interventions for children in LMICs found examples of effective strategies to reduce antibiotic consumption.^13^ The majority of interventions included were performed in hospital inpatient settings. A key finding from these reviews is the characterization of prescribers; unlike in many HICs, many primary care providers and prescribers in LMICs are not medically trained, with pharmacies or drug stores being the most used point of contact for seeking antibiotics.^11,12^ Interventions targeting the community, the largest area of antibiotic use, have greater potential for impact than those aimed purely at inpatient settings.

## Methods

This systematic review was conducted according to PRISMA 2020 guidelines and is registered on the international prospective register of systematic reviews (CRD42019116280 on PROSPERO).

We reviewed a broad range of potential interventions (both single and multifaceted) that aimed to optimize antibiotic use in children in LMIC ambulatory healthcare settings (peripheral settings), rather than hospital inpatient settings. In view of the high prevalence of self-medication and informal access to antibiotics in many LMICs, we considered a broad range of formal and informal ambulatory healthcare settings, including primary care clinics, community health posts, emergency departments, retail pharmacies, clinic pharmacies, drug shops and informal drug vendors. While we evaluated AMS interventions, we also considered interventions that had not previously been defined as stewardship by authors but had sought to optimize antibiotic use and reported an outcome of interest.

### Search strategy

We searched MEDLINE, Embase, Cochrane Central Register of Controlled Trials (CENTRAL), Cochrane Infectious Disease Group Specialized Register, Web of Science, PsycInfo and WHO library database (WHOLIS) (inception until 16 December 2024) to identify studies reporting the effectiveness of interventions to modify the use of antibiotics by infants and children with suspected infections, and to minimize the development of AMR in LMIC (as defined by the 2025 World Bank classification^14^) ambulatory healthcare settings. Ambulatory healthcare settings were defined as formal and informal peripheral healthcare settings, including primary care clinics, community health posts, emergency departments, retail pharmacies, clinic pharmacies, drug shops and informal drug vendors; both urban and rural settings were included. We examined the reference lists of articles and searched the grey literature to identify additional studies. Regional databases including African Index Medicus, Western Pacific Region Index Medicus, Latin American and Caribbean Health Sciences Literature, Index Medicus of the South East Asia Region, Index Medicus of the Eastern Mediterranean Region, Cuba Medicina, Caribbean Health Sciences Literature and the Pan American Health Organization were also searched for relevant literature.

### Outcomes

Our primary outcome was the effect of the interventions on:

1. Antibiotic prescription (or use without prescription), such as antibiotic consumption rates or percentage of cases where antibiotics are prescribed.
2. Appropriate antibiotic prescription (or use without prescription) measures, such as correct drug, dose and duration based on relevant national or international guidelines (as described by study authors).
3. Clinical outcome measures, including morbidity rates, mortality rates, infection complication rates, adverse effects of antibiotics, hospital admission rates, and suspected or proven clinical infection rates.
4. Microbiology outcome measures, including rates of infection with AMR bacteria before and after the intervention.

The range of outcomes above is necessary to be able to reach meaningful conclusions regarding effective interventions; the importance of optimizing antibiotic use is balancing the trade-offs of prompt access on one side with avoiding excess on the other.

### Inclusion criteria

Studies were eligible for review if they described interventions to optimize the use of antibiotics by infants and children (under 18 years of age) with suspected infections (diagnosed by any criteria) attending LMIC ambulatory healthcare settings. We included both single interventions (e.g. antibiotic guidelines, training, education, public reporting, delayed prescriptions and audit) and multifaceted interventions (e.g. regulatory enforcement, education, and peer influence combined). Study designs eligible for review included randomized controlled trials, non-randomized trials, controlled and non-controlled before–after studies, controlled and non-controlled interrupted time series, cohort and case control studies.

### Exclusion criteria

We excluded opinion articles, letters, reviews that did not report primary data, case series, animal studies, outbreak investigations and studies involving only surveillance of antibiotic use or AMR. We excluded papers not available in English, due to the available skillset of the authors. We excluded studies conducted exclusively in HICs as per World Bank 2024 regions.^14^ We also excluded studies conducted only in inpatient settings and studies involving inpatient and outpatient settings where separate analysis of the outpatient group was not described. Studies involving only adult patients, and studies involving children and adults where separate analysis of the paediatric population was not described were excluded. We also excluded studies involving children with significant comorbidities, such as cancer and complex medical needs. Studies with a primary focus of treating children with malaria or HIV infection were excluded as the focus of outcomes was antibiotics and bacterial infections specifically. Finally, we excluded studies of antibiotic prophylaxis, mass drug administration, vaccination or hand hygiene interventions.

### Study selection process

The eligibility assessment of titles and abstracts identified by our search was conducted independently by a team of authors (J.S.H., G.C. and E.O.) using pre-determined inclusion and exclusion criteria to dual screen each study title/abstract. This process was piloted by J.S.H. and C.C. on a representative sample of studies. Uncertainty regarding study eligibility was resolved by consensus and by consulting a third author if necessary. Full-text versions of relevant studies were independently assessed using double screening by G.C., F.C.F., J.S.H. and E.O. to obtain a list of articles suitable for evaluation using the Integrated quality Criteria for the Review of Multiple Study designs (ICROMS) tool.^15^ Two authors (of G.C., J.S.H., E.O.) evaluated the quality of each eligible publication using the ICROMS tool, with disagreements resolved as detailed above.

### Data extraction

Data were extracted by G.C., J.S.H. and E.O. using a standardized data collection tool in Microsoft Excel, built by J.S.H. Study details extracted on the form included: authors, year of publication, study design, location of the intervention (country or countries), population, sample size and setting (e.g. drug shop, primary healthcare clinic), intervention type, effect and the results of the ICROMS evaluation. We classified interventions (e.g. enabling, persuasive, structural, restrictive and decision support interventions). We also grouped studies of single and multifaceted interventions. Data synthesis included the collation and tabulation of results by intervention classification. We summarized interventions and their impact on antibiotic use and AMR in infants and children in LMIC ambulatory healthcare settings (using either relative risk, prevalence ratio or ORs as reported by each study). We did not undertake meta-analysis due to diversity of study design, interventions and outcomes.

### Risk-of-bias assessment

The risk of bias for included studies was evaluated using the ICROMS tool. Each study was assessed against mandatory criteria and scored according to study design. Disagreements between reviewers were resolved by consensus. Studies failing to meet mandatory ICROMS criteria or scoring below the threshold for their design were excluded. Of the 46 studies assessed, 23 met the ICROMS quality standards and were included in the final synthesis. This structured approach ensured that only studies with sufficient methodological rigour contributed to the review’s conclusions.

## Results

**Figure 1.**
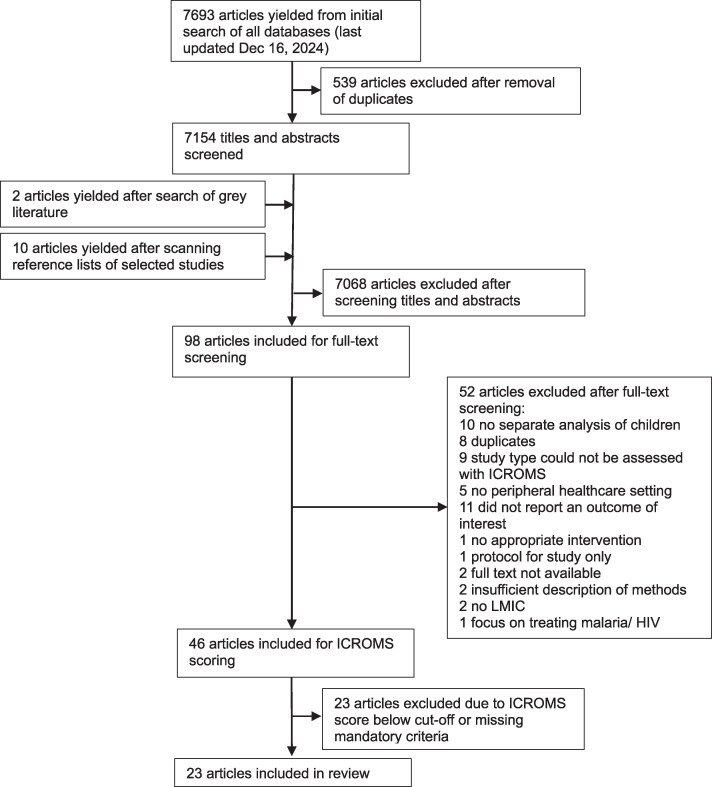
Selection of studies reporting the effectiveness of interventions to modify antibiotic use in children with suspected infections in LMIC ambulatory healthcare setting.

We identified 7154 articles on initial searching, after removal of duplicates (Figure 1). A further 12 articles were identified by searching the grey literature and scanning the reference lists of selected studies. Of 7166 articles screened, 98 were selected for full-text screening and 46 progressed to ICROMS scoring, with 23 selected for inclusion in the final review. Twenty-three studies were excluded for either missing mandatory ICROMS criteria or ICROMS scores below the threshold for the specific study design.

**Figure 2.**
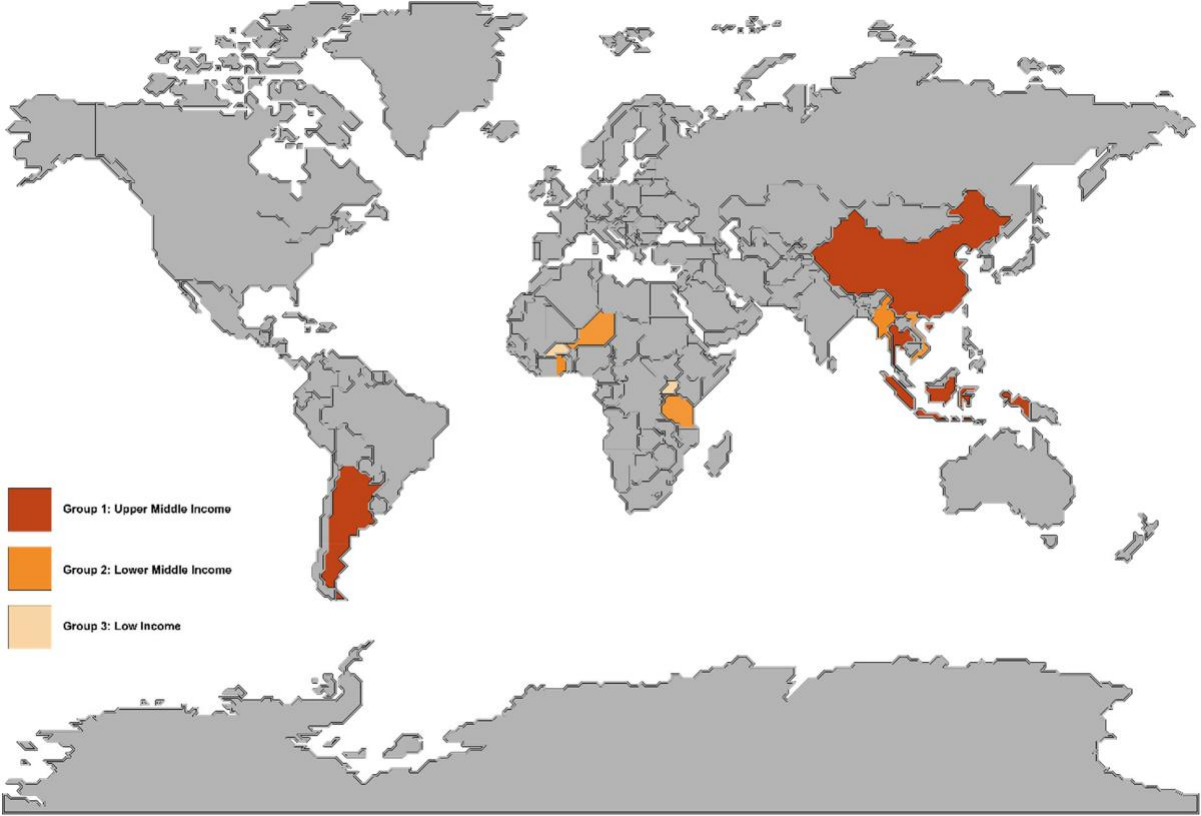
World map highlighting countries where included studies were carried out, stratified by their 2024 World Bank status.

Of the included studies, 17 (73.9%) were randomized trials and 6 (26.1%) were non-randomized trials. Two articles described one multifaceted intervention at different time points. The included studies, published between 2002 and 2024, were conducted in 11 countries, including 5 LMICs, 4 upper middle-income countries (UMICs) and 2 low-income countries (LICs) (shown in Figure 1). One study was conducted in two countries (lower middle-income and upper middle-income). Using the 2024 World Bank regions classification, 12 study sites were in East Asia/Pacific, 11 study sites were in sub-Saharan Africa and 1 study site was in Latin America/Caribbean; Figure 2 is a global depiction of countries represented within this review.

Nine studies evaluated single interventions and 14 evaluated multifaceted interventions. Of the 14 multifaceted studies, 5 used an intervention package involving diagnostic tests [C-reactive protein (CRP), WBC] and a decision support algorithm, with 4 of these also including a training/communication element. Seven studies used educational methods to target healthcare providers; these included three drug shops/pharmacies, one nurse-led healthcare centre and two country-level hospitals. These educational methods varied from both virtual and in-person training to pharmacists, drug-shop attendants, nurses and doctors as well as community awareness campaigns. Many also included other elements including subsidized drug costs, supervision and peer review meetings, clinical guidelines and certification courses.

Of the remaining two multifaceted studies, both were carried out in China: one implemented an audit with financial punishments along with feedback for prescribers, and one used a structural change of decentralizing community health centres, offering practitioners financial incentives and changing the reporting structure.

All studies reported the rates of antibiotic prescription, whilst only six studies also reported an outcome around clinical status (e.g. caregiver-reported recovery, clinical failure, warning signs checked).

Six interventions targeted primary care doctors’ antibiotic prescribing. One of these interventions also included education for caregivers. One intervention targeted the antibiotic prescribing of primary care nurses, and one intervention involved both nurses and doctors working in primary care. Two interventions involved multiple health workers, including doctors, assistant doctors, nurses, midwives, pharmacists, drug-sellers, community health extension workers and community health officers. One intervention involved ‘health workers’ in primary care facilities. Four interventions targeted the antibiotic prescribing of doctors working in paediatric hospital outpatient departments. One intervention targeted community health workers working in rural villages. Two interventions involved drug shop attendants and a community awareness campaign. One intervention involved drug sellers at registered drug shops, one intervention involved private pharmacists and one involved lead pharmacists at local pharmacies. Two studies compared the results of decision support interventions implemented by study clinicians with routine care from primary care clinicians. Included studies reaching ICROMS criteria are summarized in Table 1.

**Table 1.** Studies fulfilling ICROMS criteria

Author, year	Study design	Country	Population/setting	Sample size, duration	Intervention type	Outcome	Key findings
Randomized controlled trials							
Adjei, 2023^16^	RCT	Ghana	Participants aged 6 mo to <18 y with acute non-severe febrile illness. Shai-Osudoku and Ningo-Prampram districts; 4 public health facilities with basic laboratory services	2766 participants 12 mo	1. Pathogen-specific and non-pathogen-specific POCTs for common causes of fever 2. Clinical and diagnostic algorithm based on the results of the POCT 3. Training/communication packages for enhancing healthcare workers’ and patients’/caregivers’ adherence to prescriptions	Primary: proportion of patients with favourable clinical outcomes at Day 7 Secondary: any significant difference in proportion of antibiotics prescribed between arms	1512 patients: 761 randomized to intervention, 751 to control 76% aged <5 y and 53% male; 11% relative risk reduction of antibiotic prescription in intervention group (RR, 0.89; 95% CI, 0.79–1.01); 14% in children aged <5 y (RR, 0.86; 95% CI, 0.75–0.98); 15% in non-malaria patients (RR, 0.85; 95% CI, 0.75–0.96); 16% in patients with respiratory symptoms (RR, 0.84; 95% CI, 0.73–0.96) Majority favourable outcomes in both arms (99.7% intervention vs 99.4% control) Adherence similar between arms
Althaus, 2019^17^	RCT	Thailand, Myanmar	Children (aged 1–12 y) and adults with documented fever or history of fever; 6 primary care units in Thailand and 3 primary care clinics and 1 outpatient department in Myanmar	1201 children 14 mo	Decision support (CRP RDT)	Antibiotic prescription from Days 0–5, proportion of patients prescribed an antibiotic at CRP thresholds	Control (*n* = 402) vs CRP threshold of 20 mg/L (group A, *n* = 400) vs CRP threshold of 40 mg/L (group B, *n* = 399) Thailand: Reduction in antibiotic prescribing for group A (31.4%) (aOR 0.85; 95% CI, 0.55–1.31) and group B (26.9%) (aOR 0.68; 95% CI, 0.43–1.06) vs control (34.9%) Myanmar: Reduction in antibiotic prescribing in group A (40.8%) (aOR 0.95; 95% CI, 0.64–1.41) and B (38.4%) (aOR 0.86; 95% CI, 0.57–1.29) vs control (42.0%)
Chuc, 2002^18^	RCT	Vietnam	Children aged <5 y with uncomplicated URTI presenting to private pharmacies	58 private pharmacies; 2 y and 4 mo	1. Restrictive (regulatory enforcement) 2. Enabling (treatment guidelines, face-to-face education sessions) 3. Persuasive (peer influence)	Proportion cases where antibiotics dispensed, proportion of consultations where questions related to breathing were asked	Intervention pharmacies decrease in antibiotic prescription from 45% to 30%, Control pharmacies increased antibiotic prescriptions from 39% to 42% (*P* < 0.05)
Ciccone, 2023^19^	Cross-sectional stepped wedge CRCT	Uganda	Children aged 2 mo to 5 y presenting to CHW with fever and cough Villages (clusters) in Bugoye sub-county	15 clusters (villages) with 65 CHWs Transitioning from control to intervention condition in 5 waves of 3 clusters each and 6 total periods of follow-up, 1080 children 7 mo	Clinical algorithm (including CRP measurement) Control: Integrated Community Care Management	Primary: whether an antibiotic was given or prescribed by the CHW during the initial assessment Secondary: (i) persistent fever on Day 7; (ii) development of danger signs; (iii) unexpected visits to the CHW during the 7 day follow-up period; (iv) hospitalizations; (v) deaths; (vi) lack of perceived improvement per the child’s caregiver on Day 7; (vii) clinical failure	1220 enrolled with sufficient data; 48% in control period and 52% in intervention periods Observed percentage of antibiotics at the initial visit: 91.8% in control periods; 70.8% during the intervention periods (adjusted prevalence difference −24.6%; 95% CI, −36.1% to −13.1%) Odds of antibiotic prescription were over 80% lower in the intervention compared with the control periods (OR 0.18; 95% CI, 0.06–0.49). Frequency of clinical failure: 3.9% in control vs 1.8% in intervention (11/630; OR 0.41; 95% CI, 0.09–1.83) Lack of perceived improvement by the caregiver: control 2.1% vs intervention 3.5% (OR 1.49; 95% CI, 0.37–6.52) No unexpected visits or deaths in either group within follow-up
Do, 2016^20^	RCT	Vietnam	Children and adults aged 1–65 y with non-severe ARI at 10 primary healthcare centres. Subgroup analysis of children (1–15 y)	1028 children (510 intervention group, 518 control group) 17 mo	Decision support (CRP POCT) vs control (routine care). CRP threshold in intervention group was 10 mg/L for children aged 1–5 y and 20 mg/L for children 6–15 y	Antibiotic use within 14 d of follow-up, proportion of children receiving an immediate antibiotic prescription, antimicrobial activity in urine	Children receiving antibiotics within 14 d: 65.8% in intervention vs 76.8% in control group (OR 0.55; 95% CI, 0.4–0.75; *P* = 0.0001) Children receiving immediate antibiotic: 44.5% intervention vs 333/518 (64.3%) in control group (OR 0.39; 95% CI, 0.30–0.52; *P* < 0.0001) Children with antibiotics in urine: 30.1% intervention vs 35.5% in control group (*P* = 0.06)
Do, 2023^21^	CRCT	Vietnam	Children and adults aged 1–65 y attending 48 commune health centres with suspected non-severe ARI. Subgroup analysis of children (aged 1–15 y)	10 736 children (4901 intervention group, 5835 control group) 11.5 mo	Decision support (CRP POCT) vs control (routine care). CRP thresholds: <10 mg/L no antibiotic recommended, 10–40 mg/L antibiotic considered if high clinical concern, >40 mg/L antibiotic recommended	Proportion of patients prescribed antibiotics for ARI	Children prescribed antibiotics: 93.6% in intervention vs 97.3% in control group (aRR 0.86; 95% CI, 0.62–0.97)
Hoa, 2017^22^	CRCT	Vietnam	Children aged <5 y with ARI presenting to medical and pharmacy personnel working in public and private health facilities	2021 prescriptions, 284 HCPs 7 mo	Enabling (education, case scenario discussion, poster distribution)	Differences in improvement in knowledge and practice (antibiotic prescription for children with ARI)	Children prescribed antibiotics for mild ARI: 28% reduction in intervention group vs 3% reduction in control group Antibiotic prescriptions for severe ARIs increased from 80% to 94% in the control arm whilst reducing from 100% to 99% in the intervention arm. Improvement in HCPs’ knowledge of ARI management in intervention versus control groups
Kapisi, 2023^23^	RCT	Uganda	Febrile outpatients aged ≥1 y 3 public health facilities	Optimal sample size of 2400 was determined based on the expected relative reduction in antibiotic prescriptions of 30%, at 80% power and a significance level of 5% 12 mo	The intervention arm included a package of POCTs, a diagnostic and treatment algorithm, and training-and-communication messages	Proportion of patients who received an antibiotic prescription Proportion who had a favourable clinical outcome (defined at Day 7 as having a normal body temperature and reporting that Day 0 symptoms had improved or resolved)	Overall no difference in antibiotic prescriptions between arms (RR 1.03; 95% CI, 0.96–1.11) Size and direction of effects varied across sites and in different subpopulations. One site (Nagongera) showed a small difference between study arms (RR 1.19; 95% CI, 1.00–1.41); no difference was seen for the other 2 sites: Aduku (1.04; 95% CI, 0.93–1.16) and Kihihi (0.94; 95% CI, 0.85–1.05) For children stratified by age (<5, 5–10, and 10–15 y) in Aduku and Nagongera, larger differences in favour of more antibiotic prescription were seen, although this was not significant in all subgroups. In Kihihi, the situation was different: antibiotic prescribing was lower in children aged younger than 5 y (RR 0.76; 95% CI, 0.62–0.94) but was not significantly different in older age groups
Keitel, 2017^24^	RCT Non-inferiority trial	Tanzania	Children aged 2–59 mo with acute febrile illness presenting to outpatient clinics and health centres	3719 children (1586 in e-POCT intervention group, 1583 in ALMANACH control group, 544 routine care group) 15 mo	1. Decision support (e-POCT algorithm) 2. Decision support (ALMANACH IMCI-derived algorithm) 3. Routine care	Primary: proportion of clinical failures (severe symptoms, clinical pneumonia on/after Day 3 or persistent symptoms at 1 wk) Secondary: proportion of patients prescribed antibiotics	Clinical failures: 2.3% in e-POCT group vs 4.1% with ALMANACH (risk difference of clinical failure −1.7; 95% CI, −3.0 to −0.5) vs 4.6% with routine care Children prescribed antibiotics: 11.5% with e-POCT vs 29.7% with ALMANACH (RR 0.39; 95% CI, 0.33– 0.45) vs 94.9% with routine care
Kiemde, 2023^25^	RCT	Burkina Faso	Children from 6 to 59 mo of age with acute fever (axillary temperature ≥ 37.5°C) or history of fever within the past 7 d seen at Clinical Research Unit of Nanoro (CRUN) field station of Siglé in Burkina Faso	1176 children 12 mo	1. Control arm 2. Intervention package: RDT decisional algorithm (RDT-DA) arm, diagnostic tests and training-communication package for healthcare workers and parents/caregivers	(i) The rate of febrile cases with favourable outcomes at Day 7 in each arm (ii) The proportion of antimalarial and antibiotic prescriptions for acute febrile cases	Favourable outcomes at Day 7 between arms (intervention 99.5% and control 100%, *P* = 0.135) Antibiotic prescription lower in intervention arms than control arm (40% vs 57%) Reduced antibiotic prescription in intervention arm for the under-5s (RD −20.4%; 95% CI, −26.0% to −14.9%; *P* < 0.001), for the10–18 y age groups (RD −31.2%; 95% CI, −50.6% to −11.8%; *P* = 0.002), and the 5–10 y group (RD −2.8%; 95% CI, −8.9% to 14.4%; *P* = 0.707) Effects were pronounced in positive malaria diagnoses or respiratory conditions
Pagaiya, 2005^26^	CRCT	Thailand	Children aged <5 y with ARI and diarrhoea treated at 18 nurse-led primary health centres	9 intervention and 9 control primary health centres 6 mo	1. Enabling (guidelines for the management of ARI and diarrhoea, 3 day training course 2. Persuasive (outreach visit) 3. Control Training strategies included lectures, group discussions, role play and presentations	Proportion of children with ARI or diarrhoea who received an antibiotic	Reduction in proportion of children prescribed an antibiotic for ARI in intervention group (41.6% at baseline vs 27% at 6 mo) vs control group (26.7% to 29.5%; *P* = 0.02) Reduction in antibiotics prescribed for children with diarrhoea: intervention (84.8% to 83%) and control groups (96.8% to 94.7%; *P* = 0.3)
Tan, 2024^27^	CRCT	Tanzania	Sick children 2 to 59 mo old presenting to primary care facilities for an acute illness	25 patients in each of 9 clusters (health facilities) per arm, a total of 225 consultations in each of control and intervention arms 11 mo	ePOCT+, an electronic clinical decision support algorithm on an android-based tablet, along with associated POCTs (CRP, haemoglobin, pulse oximetry), training and mentorship ePOCT+ prompts the healthcare provider answering questions about demographics, symptoms, signs and tests	Primary: mean proportion of 14 pre-identified major IMCI symptoms and signs assessed by the healthcare provider, as observed by an external clinical research assistant Secondary: antibiotic prescription, appropriateness of antimalarial and antibiotic prescriptions, if counselling was provided	Mean proportion of signs/symptoms assessed was 46% in intervention facilities and 26% in control facilities (adjusted difference 15.1%; 95% CI, 4.8%–25.4%) Observed antibiotic prescription was 37.3% in intervention facilities and 76.4% in control facilities (aRR 0.5; 95% CI, 0.4–0.7; *P* < 0.001) Appropriate antibiotic prescription was 81.9% in intervention facilities and 51.4% in control facilities (aRR 1.5; 95% CI, 1.2–1.8; *P* = 0.003)
Rambaud-Althaus, 2017^28^	CRCT	Tanzania	Children aged 2–59 mo with an acute medical problem not related to injury presenting to 9 primary HFs	504 consultations (166 in control, 171 paper ALMANACH, 167 electronic support). 3 HFs in each arm 9 mo	Decision support (electronic ALMANACH vs paper ALMANACH vs control) All children had a second consultation by an expert to determine final treatment	Antibiotic prescription rate, antibiotics prescribed when indicated and proportion of children checked for danger signs	Antibiotic prescription rate: 25% in electronic ALMANACH group (RR 0.3; 95% CI, 0.2–0.5) vs 26% in paper ALMANACH group (RR 0.4; 95% CI, 0.2–0.6) vs 70% in control group Children in need of an antibiotic who got a prescription: 48% in electronic ALMANACH group (RR 0.5; 95% CI, 0.4–1.0) vs 36% in paper ALMANACH group (RR 0.4; 95% CI, 0.2–0.6) vs 100% in control group Proportion of children who received an assessment of danger signs: 74% in electronic ALMANACH group vs 41% in paper ALMANACH group vs 3% in control group
Shao, 2015^29^	CRCT	Tanzania	Children aged 2–59 mo with acute illness presenting to 4 PHC facilities	1465 children (844 children from 2 intervention facilities, 623 children from 2 control facilities) 7 mo	Decision support (ALMANACH) vs control	Proportion of children who received antibiotics on Day 0, proportion of children cured on Day 7	Day 0 antibiotic prescriptions: ALMANACH group (130/842, 15.4%; 95% CI, 12.9%–18.9%) Control group (525/623, 84.3%; 95% CI, 81.4%–87.1%) Proportion of children cured at Day 7: ALMANACH (97.3%) vs control (92.0%, *P* < 0.001)
Torres, 2014^30^	RCT	Argentina	Children aged 3–60 mo treated for non-severe pneumonia in outpatient clinic of a paediatric hospital	120 children (60 in intervention group, 60 in control group) 12 mo	Decision support (bacterial pneumonia score, BPS) vs control	Proportion of children who received antibiotics initially in each group, clinical outcome	Initial antibiotic prescription: BPS group (28/60) vs control (52/60), respectively (OR 0.13; 95% CI, 0.05–0.35; *P* < 0.001) Unfavourable clinical outcome in 5 patients in each group (OR 1.0; 95% CI, 0.2–3.6; *P* = 1.0)
Wei, 2017^31^	CRCT	China	Children aged 2–14 y with URTI attending outpatient departments of 25 primary care township hospitals in 2 counties	9800 prescriptions (4700 prescriptions from 12 intervention hospitals, 5100 prescriptions from 13 control hospitals)	Enabling (URTI guidelines and 2 h interactive training sessions for doctors; leaflets and a video educating caregivers about antibiotics) Persuasive (monthly peer review meetings, during which doctors’ antibiotic prescribing rates were assessed vs control	Antibiotic prescription rate (APR; proportion of prescriptions for childhood URTI that included 1 or more antibiotic)	APR 1936/2349 (82%) to 943/2351 (40%) in intervention group APR from 1922/2548 (75%) to 1782/2552 (70%) in control group Intervention effect (absolute risk reduction in antibiotic prescribing) 29% (95% CI, −42% to −16%; *P* = 0.0002)
Wei, 2019^32^	CRCT	China	Children aged 2–14 y with URTI attending outpatient departments of 14 primary care township hospitals in 1 county	8769 prescriptions (5084 prescriptions in 7 intervention hospitals, 3685 prescriptions in 7 control hospitals 18 mo after intervention developed by Wei *et al.*, 2017^32^)	Enabling (URTI guidelines and 2 h interactive training sessions for doctors; leaflets and a video educating caregivers about antibiotics) Persuasive (monthly peer review meetings, during which doctors’ antibiotic prescribing rates were assessed vs control	Antibiotic prescription rate (proportion of prescriptions for childhood URTI that included 1 or more antibiotic) at 18 mo following intervention implementation	Sustained reduction in APR in intervention hospitals in 1 county from 1171/1400 (84%) at baseline to 515/1380 (37%) at 6̴ mo and 2748/5084 (54%) at 18 mo vs no significant change in APR in control hospitals: 1063/1400 (76%) at baseline, 1084/1400 (77%) at 6 mo and 2772/3685 (75%) at 18 mo Adjusted APR between baseline and 18 mo was not significantly different to the difference in APR between baseline and 6 mo (13%; 95% CI, −7% to 33%; *P* = 0.21)
**Controlled before-and-after trials**							
Ferdiana, 2024^33^	CBA	Indonesia	Community pharmacy with pharmacists-in-charge as the participants. All community pharmacies in Semarang were invited to participate	80 community pharmacies, hence 80 community pharmacists, in each group (160 in total) 7 mo	1. Online educational sessions for pharmacists 2. Awareness campaign targeting customers 3. Peer visits 4. Pharmacy branding and pharmacist certification	Rate of non-prescription antibiotic dispensing	Preintervention rates of non-prescription antibiotic dispensing by community pharmacies: 76.3% in the participating and 84.6% in the non-participating groups (*P* = 0.005) Postintervention, rates of non-prescription antibiotic dispensing were 55.4% in the participating pharmacies and 82% in the non-participating pharmacies (*P* < 0.001) Reasons for continued non-prescription dispensing: financial motives, customer demand and the absence of a pharmacist in their outlet
Awor, 2014^34^	CBA	Uganda	Children aged <5 y with symptoms of fever or cough with fast breathing or diarrhoea at 84 private drug shops	3759 household interviews, 943 exit interviews 12 mo	Decision support (malaria RDTs and respiratory timers), structural (pre-packaged subsidized drugs) and enabling (drug shop attendant training, community awareness) vs control district	Children with suspected pneumonia (cough and fast breathing) treated with amoxicillin Overall antibiotic use (for pneumonia, fever and diarrhoea)	Proportion of children with pneumonia who received amoxicillin in the intervention group from 0/24 (0%) to 55/73 (75.3%) and in the control group from 0/8 (0%) to 12/45 (26.7%) (PR 2.8; 95% CI, 2.0–3.9) Overall antibiotic use increased from 65.1% to 73.5% in the control group and from 45% to 60% in the intervention group (PR 0.82; 95% CI, 0.69–0.97)
Kitutu, 2017^35^	CBA	Uganda	Children aged <5 y treated at 84 drug shops for uncomplicated malaria, pneumonia or non-bloody diarrhoea	981 children (497 in intervention group, 484 in control group). 61 drug shops in intervention group, 23 drug shops in control 20 mo	iCCM intervention vs control. Enabling (training, education, information and communication), structural/decision support (modified supply of medicines and diagnostics including malaria RDT and respiratory rate counters) and persuasive (monthly support supervision by study field supervisor)	Proportion of children who received appropriate treatment according to national and WHO guidelines. Secondary outcomes included the proportion of children prescribed an antibiotic	Increase in appropriate treatment in intervention group of: uncomplicated malaria: 80.2% (95% CI, 53.2%–107.2%); pneumonia symptoms: 65.5% (95% CI, 51.6%–79.4%); non-bloody diarrhoea: 31.4% (95% CI, 1.6%–61.2%) Provision of amoxicillin for suspected pneumonia symptoms: 4.8% to 93.2% in the intervention arm; 3.1% to 0% in the control arm (*P* < 0.001) Proportion of children prescribed an antimicrobial in the intervention drug shops declined during the study (linear trend slope 0.009; *P* < 0.001)
**Interrupted time series trials (controlled and non-controlled)**							
Gong, 2016^36^	NCITS	China	Children treated at paediatric hospital with separate analysis of outpatient population	19 482 191 outpatient prescriptions 23 mo	Restrictive (formulary restriction with prior authorization) and persuasive (financially punishing audit and feedback)	Proportion of antibiotic prescriptions, proportion of prescriptions containing restricted/unrestricted antibiotics and expenditure on antibiotics	Restrictive intervention: no reduction in antibiotic prescription Persuasive intervention: 59.4% reduction in proportion of outpatient prescriptions c (ᵦ = −6.3, *P* < 0.001). Reduction in both restricted (*P* < 0.001) and unrestricted antibiotics (*P* < 0.001) prescriptions
Liang, 2014^37^	CITS	China	Children aged <5 y with acute URTIs treated at community health centres (CHCs)	23 481 electronic medical records (14 189 in intervention group and 9292 in control group). 5 CHCs in intervention group, 2 CHCs in control group	Structural (governance structural reform involving changes to self-managed independent model regarding finance, personnel and employee compensation) vs control. Reforms meant that staff were no longer dependent on the volume and cost of medications for their income	Proportion of children receiving an antibiotic. Proportion of children receiving an antibiotic injection per month. Proportion of children receiving 2 or more antibiotics conditional on receiving an antibiotic per month	No significant difference in the proportion of children who received an antibiotic between the intervention and control groups (*P* > 0.05) Reduction (9.17%, *P* < 0.05) in the proportion of children receiving an antibiotic injection per month and reduction in the proportion of patients receiving 2 or more antibiotics per month (7.34%, *P* < 0.05)
**Cohort studies**							
Schmitz, 2022^38^	CS	Nigeria	Children aged 2–59 mo presenting to 89 primary healthcare (PHC) facilities with acute illness	1929 children (1021 children from 45 intervention PHC facilities, 908 children from 44 control facilities) 7 mo	Decision support (ALMANACH) vs control	Primary: caregiver-reported recovery at Day 7 Secondary: antibiotic prescription	Caregiver-reported recovery for children managed with ALMANACH vs control (aOR 2.63; 95% CI, 1.6–4.3) Proportion of children prescribed parenteral antimicrobials with ALMANACH (17.0%) vs control (9.4%; OR 1.98; 95% CI, 1.41–2.63) Proportion of children prescribed oral antimicrobials with ALMANACH (64.2%) versus control (74.4%) (OR 0.62; 95% CI, 0.5–0.76)

ALMANACH, new algorithm for managing childhood illness using mobile technology; aOR, adjusted odds ratio; APR, antibiotic prescription rate; ARI, acute respiratory tract infection; aRR, adjusted relative risk; BPS, bacterial pneumonia probability score; CBA, controlled before-and-after; CHC, community health centre; CHW, community health worker; CITS, controlled interrupted time series; CRCT, cluster randomized controlled trial; CRP, C-reactive protein; CS, cohort study; e-POCT, electronic algorithm using host biomarker point-of-care tests for the management of febrile illness in Tanzanian children; HCP, healthcare provider; HF, healthcare facility; iCCM, integrated community case management; IMCI, Integrated Management of Childhood Illness; NCITS, non-controlled interrupted time series; PHC, primary healthcare; POCT, point-of-care testing; PR, prevalence ratio; RCT, randomized controlled trial; RDT, rapid diagnostic test; RR, relative risk; URTI, upper respiratory tract infection. β = regression coefficient in the interupted time series model.

### Single interventions

#### Decision support/structural

Of the nine single intervention studies, four used a point-of-care test (POCT) CRP result as a decision support tool, three used a decision support algorithm, one used a clinical prediction rule and one used education of healthcare providers.

#### Electronic algorithms

All studies of electronic algorithms were performed in sub-Saharan Africa and evaluated the new algorithm for managing childhood illness using mobile technology (ALMANACH).^24,28,29,38^ The ALMANACH algorithm was developed from the Integrated Management of Childhood Illness (IMCI), incorporating findings of a literature review and aetiology of fever study in Tanzania. ALMANACH differs from IMCI in assessing more danger signs, advising urine dipstick testing in children <2 years with no source of infection identified, considering a diagnosis of typhoid fever in children ≥2 years with abdominal tenderness and no source for infection identified, and advising likely viral infection in febrile children with no classification at the end of the algorithm.^29,39^

In Tanzania, a study comparing ALMANACH with the standard management of children presenting to primary care facilities with acute illness, found significantly fewer children managed with ALMANACH were prescribed an antibiotic on Day 0 (15.4% versus 84.3%, *P* < 0.001).^29^ At Day 7, 97.3% of children in the ALMANACH group were cured compared with 92.0% in the control group (*P* < 0.001). While these results are impressive, they reflect a comparison of antibiotic prescribing by clinicians working within a study using ALMANACH with a control group of clinicians working routinely, without ALMANACH, so the study could not ascertain the level of compliance in using the algorithm within routine care, leaving a question over real-world effectiveness.

A subsequent cluster-randomized controlled trial (RCT) in Tanzania compared the management of children aged 2–59 months with acute illness presenting to primary care facilities randomized to three arms: (i) paper ALMANACH, (ii) smartphone version of ALMANACH, and (iii) standard practice (control).^28^ Only 26% of children in the paper ALMANACH arm and 25% in the smartphone ALMANACH arm received an antibiotic prescription compared with 70% in the control arm (*P* < 0.001). A significantly higher proportion of children managed with smartphone ALMANACH were checked for danger signs when compared with those managed with paper ALMANACH or those in the control group (74% versus 41% versus 3%, respectively). However, the authors noted that children assessed with ALMANACH were also significantly less likely to be prescribed an antibiotic when indicated than those in the control group (48% in the smartphone ALMANACH group versus 36% in the paper ALMANACH arm versus 100% in the control arm).^28^

A large observational study in Nigeria investigated the effectiveness of ALMANACH at improving the management of children aged 2–59 months with acute illness at primary healthcare facilities.^38^ The primary outcome of caregiver-reported recovery at Day 7 occurred in 849/994 (85.4%) cases with ALMANACH and 603/845 (71.4%) cases with routine care [adjusted odds ratio (aOR) 2.63; 95% CI, 1.60–4.32]. There was no significant difference in overall antimicrobial treatment between children managed with ALMANACH and those in the control group (77.1% versus 80.1%, respectively; aOR 0.67; 95% CI, 0.39–1.17). However, children managed with ALMANACH were more likely to receive parenteral antimicrobials and less likely to receive oral antimicrobials than those receiving routine care.^38^

An RCT in Tanzania compared ALMANACH with the electronic algorithm using host biomarker point-of-care tests for the management of febrile illness in Tanzanian children (e-POCTs).^24^ This smartphone-based algorithm incorporates oximetry, haemoglobin, procalcitonin and CRP. Children aged 2–59 months with acute febrile illness at nine outpatient clinics were randomized to ALMANACH or e-POCT with routine care documented in two health centres. The primary outcome of the proportion of clinical failures by Day 7 was 2.3% with e-POCT, lower than the 4.1% with ALMANACH (*P* = 0.005) and 4.6% with routine care. The proportion of children prescribed an antibiotic at Day 0 was also lower with e-POCT than ALMANACH or routine care (11.5% versus 29.7% versus 94.9%, respectively; *P* < 0.001). This non-inferiority trial was unable to assess algorithm uptake or compliance as study clinicians used electronic algorithms while routine clinicians in different clinics delivered routine care.^24^

#### Clinical prediction score

At a hospital in Argentina an RCT involving outpatients aged 3–60 months evaluated the bacterial pneumonia score (BPS), a clinical prediction rule for children with non-severe pneumonia.^30^ The BPS uses clinical findings, laboratory results and chest radiograph findings.^40^ Significantly fewer antibiotics were prescribed to children managed with BPS (46.6%) compared with those receiving routine care (86.6%), without increasing treatment failure (persistent symptoms, hospitalization, paediatric intensive care admission or death).

#### POCT

All studies of single POCT interventions used CRP; three were performed in the East Asia/Pacific region with the other being carried out in Uganda. Two randomized trials used CRP POCT to assess the need for antibiotics for children and adults presenting at primary care centres in Vietnam with non-severe acute respiratory infection (ARI).^20,21^ The authors performed subgroup analysis of the paediatric population (aged 1–15 years) involving 1028 children and found that significantly fewer children in the CRP group versus the control group used an antibiotic within 14 days of follow-up (65.8% versus 76.8%, *P* = 0.0001).^20^ There was no significant difference in the time for symptom resolution between the groups. The authors considered the possibility that clinicians treating patients in the control group may have reduced their antibiotic prescribing after experience of the clinical picture associated with low CRP.^20^ A recent cluster-RCT enabled mitigation of potential contamination between groups;^21^ this study evaluated the management of 10 736 children with mild ARI. Significantly fewer children in the CRP group (93.6%) than the control group (97.3%) were prescribed an antibiotic (adjusted relative risk 0.86; 95% CI, 0.62–0.97). Interestingly, only 14% of eligible patients (children and adults combined) in the intervention group underwent CRP testing. The authors suggested that low CRP uptake diluted the intervention effect and advised combining CRP POCT with education and guideline and policy changes at national level.^21^

One study described the use of CRP POCT in the assessment of children and adults presenting to ambulatory care settings in Myanmar and Thailand with symptoms of fever.^17^ This RCT compared a control group with two intervention groups using different CRP thresholds for antibiotic prescription (20 mg/L in group A, 40 mg/L in group B). The use of CRP POCT with a threshold of 40 mg/L significantly reduced the proportion of patients (children and adults combined) prescribed an antibiotic from Days 0–5 when compared with routine care (34% versus 39%; aOR 0.8; 95% CI, 0.65–0.98). However, subgroup analysis of the paediatric population (1–12 years) found non-significant reductions in antibiotic prescribing for children in Thailand (group A) and Myanmar (groups A and B) versus routine care. The authors hypothesized that individual randomization may have allowed contamination between groups, limiting the effect size.^17^

One study looked at adding the use of POCT CRP to the current practice (Integrated Community Care Management, iCCM) of community health workers (CHWs) visiting rural Ugandan villages when assessing children under 5 years presenting with fever and cough.^19^ This study was carried out in a stepped wedge fashion where there was a monthly switch from control to intervention in a staggered fashion across the participating villages. Regardless of which time period, CHWs would assess the child for danger signs and refer to the nearest health facility if any were found. In the control periods, if no danger signs were found, a respiratory rate was measured and if elevated for age, a course of antibiotics given. In the intervention periods CRP was measured, and if >40 mg/L antibiotics were prescribed, and if <40 mg/L symptomatic care advised. The primary outcome of percentage of children receiving an antibiotic prescription showed significant reduction in the intervention periods [control 91.8% (539/587) versus intervention 70.8% (448/633); adjusted difference −24.6%; 95% CI, −36.1% to −13.1%).^19^ After adjusting for season and area, there was an overall reduction in antibiotic prescriptions by CHWs in intervention versus control periods (OR 0.18; 95% CI, 0.06–0.49). Adherence to the algorithms was good, with only 1.6% of those with a CRP <40 mg/L in the intervention group receiving an antibiotic prescription. The authors concluded this supports expanding access to equitable healthcare by enabling those at the frontline with more access to rural populations.^19^

### Multifaceted interventions

#### Decision support/structural and enabling interventions

In Uganda, a quasi-experimental study assessed an integrated community case management (iCCM) intervention for the management of children aged <5 years presenting to private drug shops.^34^ The structural components involved subsidized drugs and free diagnostics [malaria rapid diagnostic tests (RDTs), respiratory timers and diagnostic algorithms]. The enabling component involved a 5 day training programme for drug shop attendants. Using an adapted ice manual, attendants were trained to count respiratory rates and perform RDTs before dispensing appropriate medication. A community awareness campaign promoted appropriate care-seeking with information provided at markets, public gatherings and via radio. The primary outcome was ‘appropriate treatment’ (defined as amoxicillin) of children with presentations including cough and rapid breathing. Drug shop exit interviews found improvements in the management of children with suspected pneumonia at intervention drug shops. Respiratory timer use increased by 54.8% in the intervention group compared with no change in the control group (*P* < 0.0001). Appropriate treatment of suspected pneumonia with amoxicillin increased by 75.3% in the intervention group and by 26.7% in the control group (*P* < 0.0001).^34^

Two studies in Uganda assessed integrated Community Case Management (iCCM) interventions to increase dispensing of appropriate antibiotics for children with pneumonia at private registered drug shops (similar to pharmacies) in Uganda.^34,35^ These two studies found different effects on overall antibiotic use and did not report clinical outcomes.

Three studies used the same design, implementing an intervention package of a diagnostic and treatment algorithm that determined which POCTs were carried out. The algorithm tested all patients for malaria, then separated into respiratory versus non-respiratory symptoms; those with respiratory symptoms were further categorized by factors including age and symptoms to determine which respiratory pathogens were tested. The diagnostic tests included rapid malaria test, typhoid IgM, influenza antigen A/B, respiratory syncytial virus (RSV), streptococcus A test kit, *Streptococcus pneumoniae* urinary antigen, WBC differential, urine dipstick for leucocyte esterase and nitrites, and CRP. All results were typically available in 30 minutes and used by clinicians to guide prescribing decisions. In addition, in all three studies, a week-long training and communication package was provided before enrolment; this involved education for healthcare professionals on how to communicate the prescriptions to patients, and messages for patients aimed to increase adherence to prescription.

The study in Uganda, which ran at three sites, found no overall difference in antibiotic prescription between intervention and control arms (RR 1.03; 95% CI, 0.96–1.11), with one site alone reaching marginal statistical significance (Nagongera: RR 1.19; 95% CI, 1.01–1.49).^23^ Amongst patients testing negative for malaria, antibiotic prescriptions were significantly reduced across the entire trial (RR 0.68; 95% CI, 0.63–0.75)^23^ despite previous studies suggesting introduction of a rapid malaria test does not decrease antibiotic prescriptions.

The Burkina Faso study ran at two health facilities with both an intervention and control arm; there was a significant difference between antibiotic prescriptions in two groups (intervention 40.6% and control 57.5%; RR 29.3; 95% CI, 21.8–36; *P* = <0.001) and no difference in favourable outcomes at Day 7 (99.5% in intervention arm and 100% in control arm; risk difference −0.5; 95% CI, −1.1 to 0.1; *P* = 0.135).^25^ The effect remained significant when stratified by positive and negative malaria results. Adherence to antibiotic prescriptions was higher in the intervention versus control arm (91.3% versus 87.7%; *P* = 0.02).^25^

The Ghana study ran in four health facilities; similarly to Burkina Faso there was no difference in favourable outcomes at 7 days (intervention 99.7% versus control 99.4%) and there was a significant reduction in antibiotic prescription (RR 0.89; 95% CI, 0.79–1.01).^16^ The reduction in antibiotic prescriptions was significant in those <5 years (RR 0.86; 95% CI, 0.75–0.98) and those with negative malaria tests (RR 0.85; 95% CI, 0.75–0.96).^16^

The three studies show similar trends in reductions of antibiotic prescriptions with no detriment to favourable clinical outcomes, but not typically reaching statistical significance with overall effect; many stratified groups such as age and malaria testing did produce a significant reduction.^16,23,25^

A randomized trial in Tanzania used a clinical decision support algorithm including POCTs alongside training and mentorship within primary care facilities. Its primary outcome was the number of major IMCI (Integrated Management of Childhood Illness) symptoms assessed by clinicians, with secondary outcomes including antibiotic prescription, and the appropriateness of the prescribed antibiotics.^27^ Antibiotic prescriptions were lower in intervention health facilities (adjusted risk ratio 0.5; 95% CI, 0.4–0.7; *P* < 0.001) as observed by an external researcher. There was also an increase in major IMCI signs assessed in the intervention groups (by 15%); however, overall the number of signs assessed per consultation remained low.^27^

#### Decision support/structural, enabling and persuasive interventions

A further quasi-experimental iCCM study in Uganda utilized enabling, structural/decision support and persuasive components to improve the treatment of children aged <5 years with febrile illness at private drug shops.^35^ Enabling interventions involved drug seller training and provision of information. Structural components included supply mechanisms for diagnostics (respiratory rate counters and malaria RDTs) and medicines. The persuasive intervention was monthly support supervision. Increased guideline adherence for the treatment of malaria, pneumonia and non-bloody diarrhoea (primary outcomes) was noted at intervention drug shops, including a 65.5% increase in the correct dose and duration of amoxicillin for pneumonia (*P* < 0.001).^35^ The authors described a reduction in the proportion of children prescribed any antimicrobial at intervention drug shops during the study (linear trend negative slope = −0.009; *P* < 0.001).^35^

#### Enabling and persuasive interventions

A cluster-RCT in Thailand evaluated an intervention for the treatment of children aged <5 years with ARI and diarrhoea at 18 nurse-led primary healthcare centres.^26^ Nurses attended an enabling 3 day interactive training course covering national clinical guidelines and copies of guidelines were provided. A subsequent educational outreach visit involved enabling and persuasive components, with a discussion of guidelines, barriers to guideline use, patient record reviews and feedback on clinical management. The intervention achieved a significant reduction in antibiotic prescriptions for children with ARI (42% to 27%, *P* = 0.022) but no improvements in antibiotic prescriptions for diarrhoea.^26^

Two large studies from China examined the impact of a multifaceted intervention on antibiotic prescribing for children aged 2–14 years with upper respiratory tract infections (URTIs) treated at primary care township hospitals.^31,32^ The authors developed clinical guidelines and an interactive training session, deploying communication skills and roleplay sessions to enable doctors to use the guidelines, to correctly diagnose children with viral URTIs and to explain appropriate treatment to caregivers. Leaflets provided by doctors and a video in hospital waiting rooms educated caregivers about appropriate antibiotic use. The persuasive intervention involved monthly peer-review meetings, with assessments of doctors’ antibiotic prescribing rates. A cluster-RCT at 25 primary care hospitals showed a significant reduction in the proportion of prescriptions for childhood URTI that contained one or more antibiotics (from 82% at baseline to 40% at 6 months in the intervention group versus 75% to 70% in the control group; *P* < 0.0001).^31^ Subsequently, a follow-up study in 14 primary care hospitals 18 months post intervention found that although the antibiotic prescription rate in intervention hospitals had rebounded to 54%, it remained significantly lower than baseline.^32^

A pre-post quasi-experimental study in Indonesia targeted community pharmacists with a 7 month intervention including online education, an awareness campaign for customers, peer visits and a certification programme for the involved pharmacists.^33^ The rate of non-prescription antibiotic dispensing decreased in participating pharmacies by 20.8%, compared with a 2.3% decrease across the same time period in non-participating pharmacies (*P* < 0.001).^33^ The qualitative portion of the study revealed many factors influencing continued non-prescribed antibiotics within the intervention groups, including pressure from pharmacy owners to maximize sales, fear of losing customers if refused and customer persistence.^33^

#### Restrictive and persuasive interventions

In China, a multifaceted AMS intervention with restrictive and persuasive components was implemented at a paediatric hospital.^36^ Initially formulary restriction with prior authorization was introduced before the addition of financially punishing audit and feedback. An uncontrolled observational study found no significant reduction in outpatient antibiotic prescribing with formulary restriction and prior authorization alone. The implementation of financially punishing audit and feedback was associated with a 59.4% reduction in outpatient antibiotic prescriptions (*P* < 0.001).^36^ Clinical and microbiological outcomes were not evaluated.

#### Restrictive, enabling and persuasive interventions

A cluster-RCT in Vietnam investigated an intervention to improve practice at 58 private pharmacies.^18^ The restrictive intervention was regulatory enforcement, involving inspections by the health bureau and an explanation of drug regulations. The enabling intervention involved the development of pharmacy treatment guidelines and in-person educational sessions for pharmacy staff. The persuasive intervention involved peer influence, with appointed leaders attending a seminar before disseminating a peer influence strategy to pharmacy staff and holding regular meetings to discuss appropriate practice. The management of uncomplicated upper respiratory tract infections (URTI) in children aged <5 years was assessed using a simulated client model. The percentage of children with mild ARI who received an antibiotic in intervention pharmacies decreased from 45% to 30%, while increasing in control pharmacies from 39% to 42% (*P* < 0.05).^18^

#### Enabling interventions

A large cluster-RCT assessed the impact of a multifaceted intervention targeting antibiotic prescribing by all medical and pharmacy personnel in a district of Vietnam.^22^ Enabling techniques, including education, case scenario discussion and poster distribution, were used to improve the knowledge and practice of healthcare providers (HCPs) treating children aged <5 years with ARIs. In the intervention group, a significant improvement in HCPs’ knowledge of ARI management was associated with a 28% reduction in children prescribed antibiotics for mild ARI compared with a 3% reduction in the control group.^22^ The impact of the intervention on clinical outcomes was not assessed.

#### Structural interventions

A study in China evaluated the impact of regional governance structural reform, involving changes to finance and employee compensation, on the treatment of children aged <5 years with URTIs at community health centres.^37^ Although there was no significant difference in the proportion of children who received an antibiotic between the intervention and control groups (*P* > 0.05), significant reductions in the proportion of children receiving an antibiotic injection (9.17%, *P* < 0.01) and the proportion of children receiving two or more antibiotics (7.34%, *P* < 0.05) were noted in the intervention group.^37^

**Figure 3.**
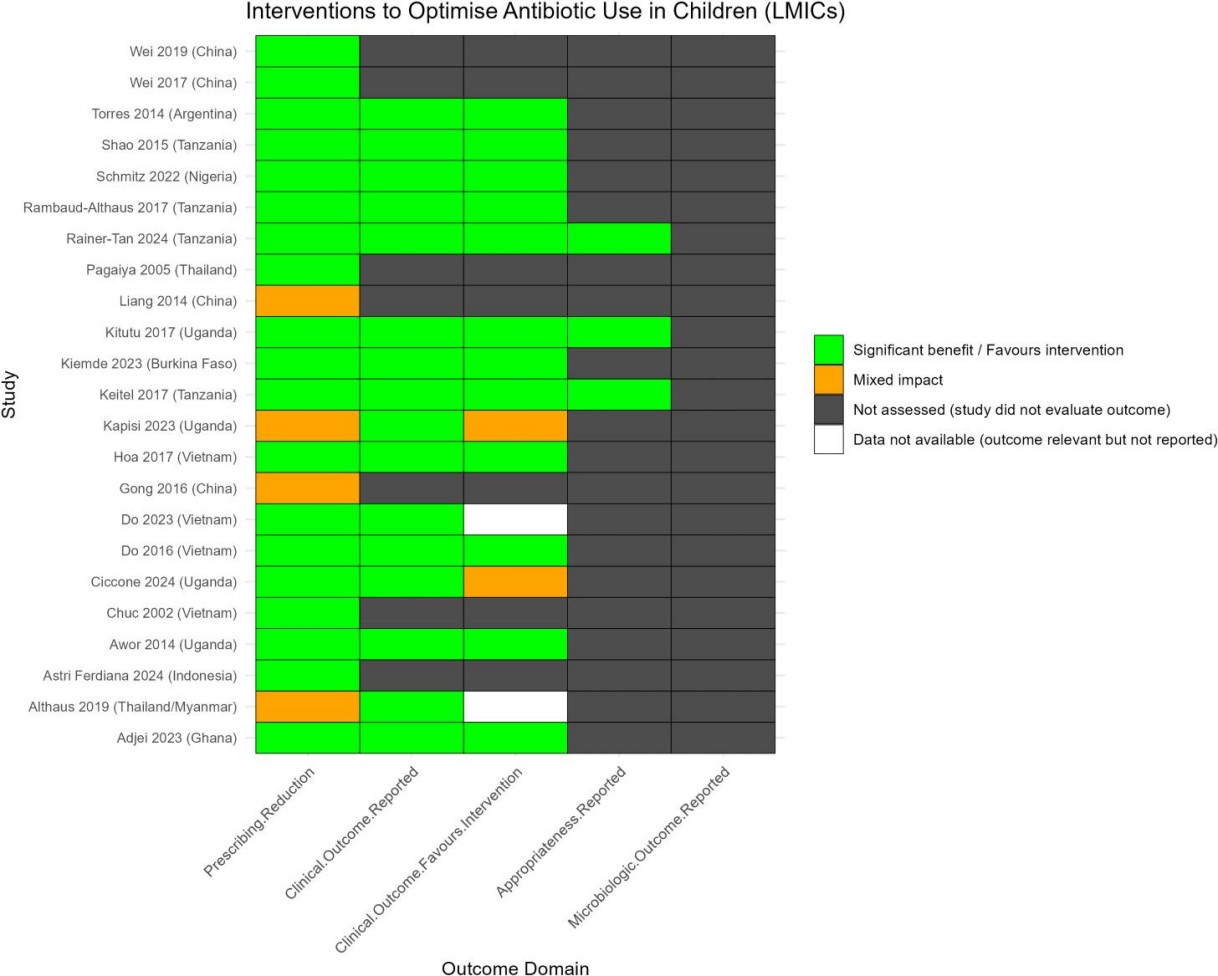
Heat map of included studies’ (*n* = 23) reported outcomes of interventions to optimize antibiotic use in children attending ambulatory healthcare settings in LMICs.

Figure 3 provides a visual representation of the outcomes reported by each included study; Tables S1–S3 (available as Supplementary data at *JAC-AMR* Online) categorize studies by intervention type and report key findings.

## Discussion

This systematic review demonstrates that interventions to optimize antibiotic use in children in LMIC ambulatory healthcare settings have shown variable but generally modest reductions in prescribing rates. The most consistent effects were observed in multifaceted approaches that combined point-of-care diagnostics, decision-support algorithms, and provider training.^16–23^ These interventions reduced unnecessary prescriptions for febrile and respiratory illnesses, while maintaining favourable clinical outcomes, suggesting that stewardship can be achieved without compromising patient safety.

However, only a minority of studies assessed appropriateness of prescriptions or microbiological outcomes, limiting conclusions about whether reductions aligned with guideline-based care or contributed to antimicrobial resistance mitigation. For example, the ALMANACH algorithm in Tanzania reduced prescribing but appeared to under-prescribe antibiotics for children who required them, raising concerns about under-treatment.^24^ This highlights the need for algorithms to be adapted to local epidemiology and supported by adequate diagnostic infrastructure to avoid unintended harm.

Educational interventions targeting pharmacists and drug sellers—who are often the primary source of antibiotics in LMICs—were effective in reducing inappropriate dispensing and improving provider knowledge.^18,22^ Structural reforms in China, such as decentralization and financial incentives, also influenced prescribing behaviour, though their generalizability to other LMICs is uncertain.^27^ The broad classification of LMICs encompasses countries with vastly different economic and healthcare infrastructures; for instance, China’s inclusion within this category may disproportionately influence pooled findings, as its health system capacity and regulatory frameworks differ substantially from those of lower-income settings.

Given China’s unique health system context, separating its findings from other LMICs in future analyses may help clarify its disproportionate impact; similarly for all countries the setting should be taken into context when applying findings to other LMICs.

Clinical outcomes were generally favourable across intervention and control groups, indicating that reduced prescribing did not compromise recovery.^16,19,20^ Nevertheless, the lack of consistent reporting on appropriateness and microbiological endpoints remains a major limitation. Future studies should prioritize these outcomes, alongside feasibility and implementation factors, particularly in settings with limited diagnostic capacity.

These findings align with WHO AMS guidance, which emphasizes context-specific, system-integrated strategies.^41^ Although RCTs provide robust evidence, well-designed before–after studies may be more feasible in resource-limited settings. Additionally, vaccine coverage—particularly for pneumonia and malaria—should be considered in stewardship planning, as improved immunization reduces infection burden and antibiotic demand.^2^

### Limitations and future research

This systematic review was constrained by heterogeneity in study design, intervention types, and outcome reporting, which precluded meta-analysis and limited comparability across settings. Few studies assessed prescription appropriateness or microbiological outcomes, restricting conclusions about long-term antimicrobial resistance impact. Moreover, most interventions targeted formal healthcare facilities, overlooking the substantial antibiotic use occurring in informal drug stores and unregulated pharmacies—settings that account for a large proportion of community antibiotic access in LMICs. Future research should prioritize context-specific AMS strategies that engage these informal providers, evaluate their prescribing behaviours, and explore scalable models for integrating them into national AMS programmes.

### Conclusions

The current evidence base for interventions to optimize antibiotic use in children in LMIC ambulatory healthcare settings is limited, with available evidence suggesting reduced antibiotic prescription rates from a broad range of interventions, but often without demonstrating clinical non-inferiority or a measure of appropriateness for the prescriptions. Effective stewardship in LMIC ambulatory settings requires adaptable, multi-component interventions that balance access with rational use, incorporate rapid diagnostics, and evaluate both clinical and appropriateness outcomes. Strengthening regulatory frameworks, provider training, and community engagement will be essential to sustaining impact. The studies that do report clinical outcomes are reassuring that infrequent negative effects are found; no studies report the impact of interventions on AMR infections. Future studies with robust study designs that include implementation research are needed to inform strategies to optimize antibiotic use and clinical outcomes in informal and formal ambulatory healthcare settings.

## Supplementary Material

dlag109_Supplementary_Data

## Supplementary data

Tables S1–S3 are available as Supplementary data at *JAC-AMR* Online.
