# Supplementary material for: The impact of interventions to modify antibiotic use in children with suspected infections in ambulatory healthcare settings in LMICs: a systematic review
Source: JAC Antimicrob Resist. 2026 Jun 13;8(3):dlag109. doi: 10.1093/jacamr/dlag109 (PMC13263530; doi:10.1093/jacamr/dlag109)
Supplement: dlag109_Supplementary_Data [file dlag109_supplementary_data.docx]

Appendix 1: Supplementary Tables detailing studies by intervention

**Table 1: Single Intervention Studies Summary:**

| **Intervention Type** | **Number of Studies** | **Description** |
| --- | --- | --- |
| POCT CRP | 4 | Used CRP levels to guide antibiotic prescribing decisions |
| Decision Support Algorithm | 3 | Electronic or paper-based clinical algorithms (e.g., ALMANACH) |
| Clinical Prediction Rule | 1 | Bacterial Pneumonia Score (BPS) used in Argentina |
| Education of Healthcare Providers | 1 | Training-based intervention |

**Multiple Intervention Studies Summary**

**Table 2: Electronic Algorithm Studies (ALMANACH & e-POCT)**

| **Country** | **Study Design** | **Intervention Arms** | **Antibiotic Prescription (%)** | **Clinical Outcome (Day 7)** | **Key Findings** |
| --- | --- | --- | --- | --- | --- |
| Tanzania | RCT | ALMANACH vs Routine Care | 15.4% vs 84.3% | 97.3% vs 92.0% | Significant reduction in antibiotic use; high cure rate with ALMANACH |
| Tanzania | Cluster RCT | Paper ALMANACH, Smartphone ALMANACH, Control | 26%, 25%, 70% | Not reported | Danger signs checked more often with smartphone ALMANACH |
| Nigeria | Observational | ALMANACH vs Routine Care | 77.1% vs 80.1% | 85.4% vs 71.4% | Higher recovery with ALMANACH; shift from oral to parenteral antimicrobials |
| Tanzania | RCT | e-POCT vs ALMANACH vs Routine Care | 11.5% vs 29.7% vs 94.9% | 2.3% vs 4.1% vs 4.6% | e-POCT showed lowest failure rate and antibiotic use |

T**able 3: Point-of-Care Testing (CRP POCT)**

| **Country/Region** | **Study Design** | **Population** | **Antibiotic Prescription (%)** | **Key Findings** |
| --- | --- | --- | --- | --- |
| Vietnam | RCT | 1,028 children | 65.8% (CRP) vs 76.8% (Control) | Significant reduction; no difference in symptom resolution |
| Vietnam | Cluster RCT | 10,736 children | 93.6% (CRP) vs 97.3% (Control) | Low CRP uptake (14%) diluted effect; recommended policy integration |
| Myanmar & Thailand | RCT | Children & adults | 34% (CRP 40mg/L) vs 39% (Control) | No significant reduction in children; possible contamination between groups. |
| Uganda | Stepped wedge trial | Children < 5 years | 70.8% (Intervention) vs 91.8% (Control) | Strong reduction; high adherence; supports rural healthcare access |
